# Isolation and genome sequence annotation of SallyK, a newly discovered cluster EG bacteriophage infecting *Microbacterium*

**DOI:** 10.1128/MRA.00943-23

**Published:** 2023-11-22

**Authors:** Paige R. Wood, Emily A. Sullivan, Rylee T. Mathison, Sariah M. Hepworth, R. Scott Graves, Breanna Danklefsen, Omer Almahie, Carlos Serna, Dylan J. Hunt, Reganne Brewster, Skylar Bartholomew, Jack F. Shurley, Anna S. Grinath, Michael A. Thomas

**Affiliations:** 1 Department of Biological Sciences, Idaho State University, Pocatello, Idaho, USA; Loyola University Chicago, Chicago, Illinois, USA

**Keywords:** bacteriophage, *Microbacterium*, SEA-PHAGES

## Abstract

Discovered from soil in a flower planter in Pocatello, Idaho and using *Microbacterium foliorum*, SallyK is a lytic bacteriophage with a siphovirus morphology. It has a 62,883 bp-long genome with 103 putative genes. Based on gene content similarity to actinobacteriophages, SallyK is assigned to cluster EG.

## ANNOUNCEMENT

**Fig 1:**
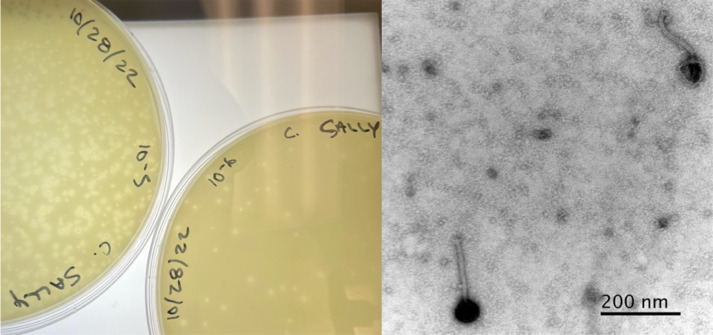
Characterization of bacteriophage SallyK. (Left) SallyK forms small and slightly cloudy plaques with a granular turbid center. (Right) Siphovirus morphology of SallyK with a capsid diameter of approximately 65 nm and a tail length of approximately 150 nm. Image produced by a Zeiss EM900 TEM with an accelerating voltage of 80 kV and uranyl acetate negative staining. 20–30 virions were measured.

As *Microbacterium* is known to contaminate fresh produce and dairy products (1), identifying bacteriophages that can kill *Microbacterium* is an important step in the development of strategies to control contamination and foodborne illnesses. Here, we describe a lytic bacteriophage, SallyK, named after the discoverer and isolated on 2 September 2021 from soil collected from a flower planter at Idaho State University in Pocatello, ID (Global Positioning System coordinates: 42.867222°N and 112.429167°W). Following established isolation procedures (2, 3), the phage was isolated by suspending soil in PYCa liquid media, centrifugation of the soil mixture, and supernatant filtration through a 0.22 µm filter. The filtrate was inoculated with *Microbacterium foliorum* NRRL B-24224. After incubation with shaking at 20°C for 48 h, an aliquot of the culture was filtered and plated in PYCa top agar with *M. foliorum*, then incubated at 20°C for 48 h. The resulting plaques were small and slightly cloudy, with a granular turbid center (Fig. 1). Plaques were purified through three additional rounds of plating. A negative stain (uranyl acetate) transmission electron micrograph of SallyK revealed a siphovirus (VS1) morphology with a long tail (Fig. 1).

SallyK DNA was extracted from lysate using a Promega Wizard DNA Clean-Up kit and prepared for sequencing using an NEBNext Ultra II FS kit. DNA was sequenced using a shotgun sequencing approach on an Illumina MiSeq with v3 reagents, resulting in 403,650 single-end 150 bp reads. Raw reads were trimmed and assembled with Newbler v2.9 using default parameters, yielding a single contig with 913-fold coverage. Consend v29 was used to check for completeness and accuracy and determine to phage termini, both using default settings (4–6). SallyK has a genome length of 62,883 bp with a direct terminal repeat of 219 bp and GC content of 67.0%, slightly lower than its host (68.7%) (7). SallyK was assigned to the phage cluster EG based on gene content similarity above a 35% threshold to other phages in the Actinobacteriophage database (8, 9).

The genome of SallyK was automatically annotated using DNAmaster v5.23.6 (http://cobamide2.bio.pitt.edu) and PECAAN (http://discover.kbrinsgd.org), followed by manual refinement of start sites using Genemark v2.5 (10), Glimmer v3.0 (11), and Starterator (http://phages.wustl.edu/starterator/). Phamerator v454 (12) was used for synteny analysis. HHPRED v2.0 (13) searches against PDB mmCIF70, Pfam-A, and NCBI Conserved Domain databases and BLASTp v2.13.0 (14) searches against NCBI and Actinobacteriophage non-redundant databases were used to predict putative gene functions. No tRNAs were identified using Aragorn v1.2.41 (15) and tRNA-SE v2.0(16). Using default parameters for all software, this annotation process yielded 103 candidate protein-coding genes for SallyK, 38 of which were assigned putative functions.

Consistent with other EG phages, SallyK encodes for a fused minor capsid MuF-like protein and capsid maturation protease, while the gene encoding for its major tail protein is located upstream of head-to-tail connector proteins. Similarly, no immunity repressor or integrase functions could be identified, consistent with the lytic lifecycle of cluster EG phages (17). Encoded by gp58 is MazG-like nucleotide pyrophosphohydrolase, shown to deplete the starvation alarmone (*P*)ppGpp, preventing bacterial cells from entering starvation mode (18) and thus providing the phage more time to replicate before host death.

## Data Availability

GenBank and SRA accession numbers for SallyK are OR159650 and SRX19690854, respectively.
